# Establishment of an N-Glycan Profiling Method for Three ERT Enzymes Used in Gaucher Disease Therapy

**DOI:** 10.3390/molecules31111904

**Published:** 2026-06-01

**Authors:** Jinliang Chen, Xinyue Hu, Lyuyin Wang, Kaixin Xu, Jing Li, Yingwu Wang, Chenggang Liang

**Affiliations:** 1 School of Life Science, Jilin University, Changchun 130012, China; chenjl23@mails.jlu.edu.cn; 2 National Institutes for Food and Drug Control, Beijing 100061, China; huxinyue@nifdc.org.cn (X.H.); wanglvyin@nifdc.org.cn (L.W.); li_jing@nifdc.org.cn (J.L.); 3 School of Life Science and Technology, China Pharmaceutical University, Nanjing 210009, China; 19850856619@163.com

**Keywords:** glycan profiling, N-glycosylation, mass spectrometry, recombinant human glucocerebrosidase, Gaucher disease, phosphorylated glycans

## Abstract

N-glycosylation, particularly terminal mannose exposure, is a critical quality attribute affecting macrophage targeting and the clinical efficacy of enzyme replacement therapy for Gaucher disease. This study developed a universal, sensitive, and quantitative method to compare the N-glycan profiles of three recombinant human glucocerebrosidase products from different expression systems: imiglucerase, velaglucerase alfa, and velaglucerase beta. Using 2-aminobenzamide labeling combined with HILIC-UPLC-FLD and high-resolution mass spectrometry, an N-glycan profiling platform was established. A multidimensional calibration system integrating retention time, glucose unit values, and mass-to-charge ratios was constructed, and collision-induced dissociation tandem MS was used to identify isomers and phosphorylated glycans. The method showed good specificity, linearity, precision, and accuracy. Glycan profiling revealed clear product-dependent differences: imiglucerase was enriched in core-fucosylated Man3 structures, velaglucerase alfa was dominated by Man9 and contained more phosphorylated and sialylated glycans, whereas velaglucerase beta showed a highly homogeneous Man5 profile. These findings demonstrate how distinct manufacturing strategies shape glycosylation patterns and provide a basis for biosimilar development and comparability assessment.

## 1. Introduction

Gaucher disease (GD) is an autosomal recessive lysosomal storage disorder caused by biallelic mutations in the GBA1 gene [1]. GBA1 encodes lysosomal acid β-glucocerebrosidase (glucocerebrosidase, GCase), which hydrolyzes glucosylceramide (GlcCer) into glucose and ceramide [2]. Deficient GCase activity results in progressive GlcCer accumulation in the monocyte–macrophage system and the formation of lipid-laden Gaucher cells, leading to multi-organ manifestations such as hepatosplenomegaly, anemia, thrombocytopenia, and skeletal involvement [3,4]. Since the clinical introduction of enzyme replacement therapy (ERT), exogenous supplementation of functional GCase has become a major therapeutic strategy for type I GD [5]. The therapeutic performance of recombinant human glucocerebrosidase (rhGBA) depends not only on catalytic activity but also on efficient macrophage recognition and cellular uptake, which are strongly influenced by N-glycan structural features, particularly terminal mannose exposure [6,7].

rhGBA is a glycoprotein containing four N-glycosylation sites, and its glycan profile is therefore a critical quality attribute (CQA) that may influence structural stability, biodistribution, receptor recognition, cellular uptake, and product consistency [8]. Different manufacturing strategies have been used to generate macrophage-targeted glycan structures. Imiglucerase is produced in Chinese hamster ovary (CHO) cells and requires ex vivo glycosidase trimming to expose mannose-rich termini [9,10,11,12]. Velaglucerase alfa is produced in the human fibrosarcoma cell line HT-1080, where kifunensine is used to preserve high-mannose glycans [13,14]. Velaglucerase beta is produced using a glycoengineered CHO cell platform in which GnT1-mediated glycan maturation is interrupted, resulting in high-mannose-type glycans with exposed mannose residues [15,16]. These distinct expression and processing strategies make rhGBA products an appropriate model for evaluating how manufacturing approaches shape N-glycan heterogeneity.

Released N-glycan analysis of rhGBA products remains analytically challenging. Glycan profiles are often highly heterogeneous and may include high-mannose, fucosylated, sialylated, phosphorylated, and GlcNAc-capped species. In addition, structurally related glycans may share the same monosaccharide composition or nominal mass but differ in branching, linkage arrangement, terminal modification, or isomeric configuration. Therefore, accurate mass information alone is often insufficient for confident structural assignment. Reliable glycan profiling requires an integrated strategy combining efficient glycan release and labeling, chromatographic separation of hydrophilic and isomeric glycans, fluorescence-based relative quantification, glucose unit (GU)-assisted retention calibration, high-resolution mass spectrometry (HRMS), and tandem MS confirmation for ambiguous or modified glycans [17].

For glycoprotein therapeutics, such analytical complexity is directly relevant to quality control. Product-specific glycans may lack authentic reference standards; minor glycoforms can be difficult to quantify reproducibly; and small shifts in glycan distribution may reflect changes in cell substrate, culture conditions, enzymatic processing, or purification [18,19,20]. Thus, N-glycan profiling methods used for rhGBA products should not only identify major glycans but also provide a robust framework for relative quantification, comparability assessment, batch-to-batch consistency evaluation, and monitoring of mechanism-related glycan attributes [21,22,23].

In this study, we established an integrated N-glycan profiling workflow for three rhGBA ERT products, including imiglucerase, velaglucerase alfa, and velaglucerase beta. The novelty of this work does not lie in the use of 2-aminobenzamide (2-AB) labeling alone but in the product-oriented combination of optimized hydrophilic interaction ultra-performance liquid chromatography with fluorescence detection and high-resolution mass spectrometry (HILIC-UPLC-FLD-HRMS), GU-assisted retention calibration, accurate-mass matching, an expanded rhGBA-relevant glycan library, CID-MS/MS confirmation of selected ambiguous structures, and conversion of complex glycan profiles into a terminal-mannose-related quality index [24,25,26,27]. Using this workflow, we systematically compared product-dependent N-glycan distributions and evaluated their relevance for structural characterization and quality-control applications of rhGBA products.

## 2. Results and Discussion

### 2.1. Method Development and Validation for N-Glycan Release, Separation, and Detection

To meet the analytical requirements for quality control and structural comparability assessment of therapeutic rhGBA, we established an N-glycan profiling method that integrates structural identification with relative quantification. This approach enables comprehensive evaluation of glycan composition and relative abundance, thereby providing technical support for monitoring key CQAs. Since the terminal mannose content, glycan heterogeneity, and potential phosphory-lated glycoforms are closely associated with receptor recognition and biological function of rhGBA products, the method was designed not only to quantify major glycan species but also to support the identification of structurally related glycoforms. For method development, a workflow was selected that offers short processing time, minimal sample loss, and a high signal-to-noise ratio, while maintaining sufficient compatibility with routine quality control analysis.

The workflow of the developed method is as follows: PNGase F was used to specifically cleave N-glycosidic bonds, releasing N-glycans from the protein backbone under mild enzymatic conditions. Proteins and other high-molecular-weight components were subsequently removed by ultrafiltration, and the released glycans were vacuum-dried to reduce matrix interference before derivatization. The purified glycans were then labeled with the fluorescent tag 2-AB, which not only significantly enhanced detection sensitivity but also improved chromatographic separation during HILIC analysis. After UPLC separation, glycans were detected sequentially by FLD and high-resolution Q-TOF MS, allowing relative quantification based on fluorescence response and structural assignment based on accurate mass information. Following preliminary MS-based identification, the LC gradient and column temperature were further optimized so that each retention time corresponded to a distinct rhGBA glycoform, thereby improving peak resolution and reducing potential interference from co-eluting species. In addition, the use of GU calibration and MS confirmation increased the reliability of glycan assignment, particularly for isomeric and phosphorylated structures. The method was confirmed to be applicable to the three commercially available rhGBA products. Using velaglucerase beta as a representative, specificity, linearity, repeatability, intermediate precision, and accuracy were evaluated; the results are summarized in Table 1.

Overall, the validation results demonstrated that the developed N-glycan profiling method provides excellent specificity, linearity, repeatability, intermediate precision, and accuracy. Retention times were highly stable, and quantitative bias was well controlled. The method is suitable for N-glycan compositional assessment and structural profiling of rhGBA and can be applied to lot-to-lot consistency evaluation, process-change comparability studies, and stability testing. Thus, it serves as a robust analytical platform for investigating rhGBA glycan heterogeneity and structure–function relationships.

### 2.2. Glycoform Identification

A GU standard curve was generated using a 2-AB dextran calibration ladder to verify the established profiling method. By integrating GU values with *m*/*z* information, N-glycans from the three rhGBA products were partially assigned. Because the UNIFI glycan library primarily targets antibodies, its limited coverage restricted automated matching for several peaks. To identify unmatched chromatographic peaks, plausible *m*/*z* values were calculated, and the UNIFI glycan library was extended by importing corresponding structures. This allowed automated one-click identification with mass-error reporting. For example, in positive-ion mode, the monoisotopic mass of the singly charged, protonated 2-AB-labeled pentasaccharide core is 1355.5095. The monoisotopic masses of free monosaccharides are: mannose/galactose 180.0634, GlcNAc 221.0899, fucose 164.0685, sialic acid 309.1060, and phosphate 79.9663. As one molecule of water (18.0106 Da) is lost during glycosidic bond formation, the addition of one mannose residue increases mass by 162.0528 Da. Mass increments for other residues and structural units are summarized in Table 2.

Before each analytical sequence, one injection of the 2-AB dextran calibration ladder was performed under the same chromatographic conditions as the sample analysis. The retention times of the dextran ladder peaks were processed in UNIFI software (version 2.0.194.0) to generate a fifth-order polynomial calibration curve for conversion of retention time into glucose unit (GU) values.

**Figure 1:**
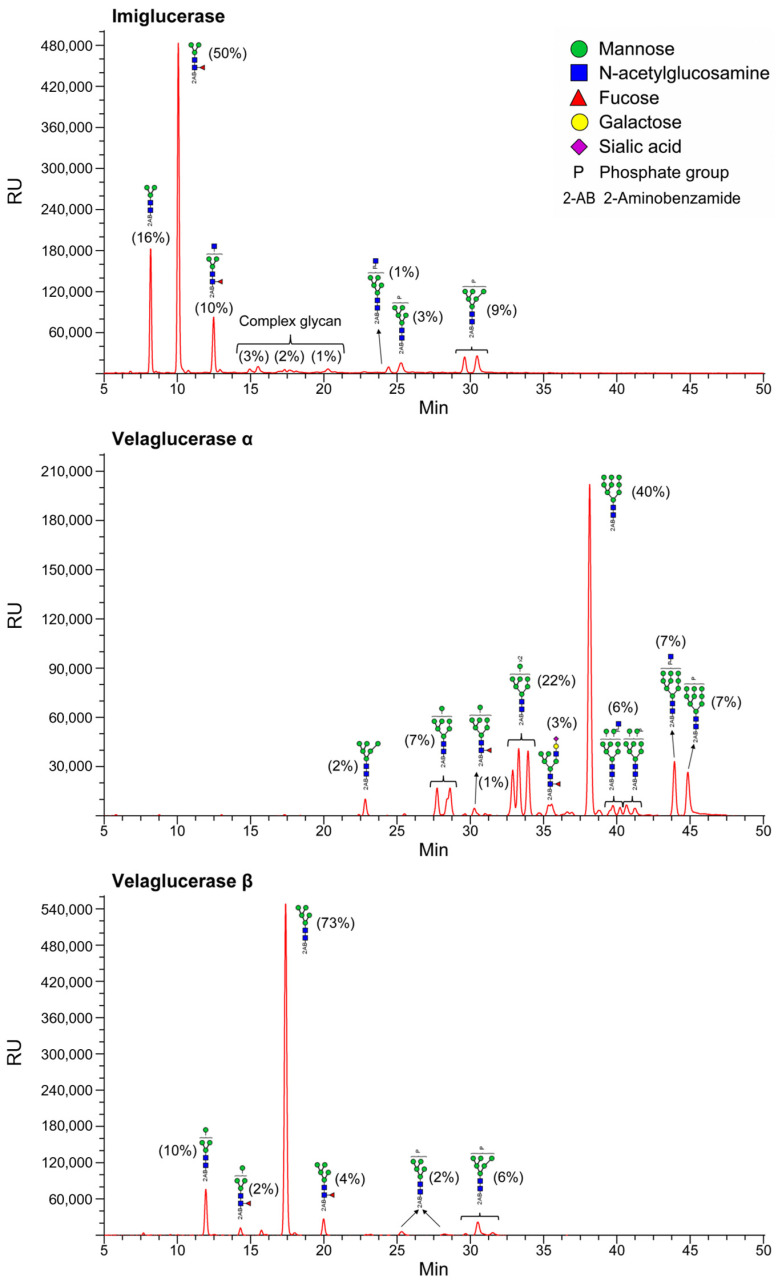
Glycoform identification of the three rhGBA products.

This approach is consistent with the Waters 2-AB dextran ladder workflow, in which GU values are calculated by fitting a fifth-order polynomial curve to the dextran ladder and then assigning GU values to sample peaks according to their retention times. The experimentally determined fifth-order polynomial calibration equation was Log(y) = 8.06 × 10^−9^x^5^ − 1.34 × 10^−6^x^4^ + 8.68 × 10^−5^x^3^ − 0.0028x^2^ + 0.0541x + 0.359, where x represents retention time and y represents the GU value. The calculated GU values were used as retention-based descriptors to support N-glycan peak assignment. Extracted ion chromatograms were generated to obtain *m*/*z* values for each chromatographic peak, and charge states were used to determine molecular masses. In parallel, monosaccharide compositions were inferred from mass increments, allowing initial glycoform assignments. The proposed N-glycan structures were confirmed through an integrated evaluation of GU values, retention behavior, accurate mass, charge state, and MS/MS evidence where available. After manual verification, results were imported into UNIFI for library expansion and structural refinement. The final annotated glycan chromatograms and glycoform statistics for the three rhGBA products are shown in Figure 1 and Table 3, respectively.

Overall, high-mannose glycans predominated in all three rhGBA products, but their distributions differed markedly. Imiglucerase consisted primarily of F(6)Man3 (~50%), with fucosylated species accounting for ~60%. Minor phosphorylated high-mannose glycans were also detected. Compared with the other products, imiglucerase exhibited shorter mannose chains, consistent with the intended ex vivo exoglycosidase trimming designed to shorten glycans and enhance TME. It also displayed the highest level of fucosylation among the three products, which may influence mannose receptor binding affinity. Velaglucerase alfa was dominated by Man9 (~40%) and contained other long-chain high-mannose structures (Man6–Man8), resulting from kifunensine-mediated inhibition of glycan processing that preserves extended mannose chains. A significant proportion of phosphorylated high-mannose glycans was also detected, consistent with lysosomal-enzyme-associated glycoforms. Additionally, a small fraction (~3%) of sialylated glycans was present, which may slightly reduce macrophage uptake efficiency but could modestly prolong in vivo circulation time. Velaglucerase beta displayed predominantly Man5 (~73%), with remaining glycoforms mainly ranging from Man4 to Man6. Only trace levels of fucosylation and phosphorylation were observed, making it the product with the highest proportion of a single glycoform (Man5). Detailed glycan species and their percentages are shown in Table 3.

Based on these glycan profiling results, the terminal mannose exposure (TME) index was further calculated to convert the complex multi-peak N-glycan distribution into a single mechanism-related quality-control parameter. Unlike simple reporting of individual glycan percentages, the TME index integrates both the relative abundance of each glycoform and the number of exposed terminal mannose residues, thereby providing a more intuitive assessment of mannose-related targeting potential. Therefore, TME can serve as a practical supplementary quality attribute for comparing products manufactured using different glycoengineering strategies, monitoring batch-to-batch consistency, evaluating process changes, and identifying potential glycosylation drift during development or routine quality control. However, TME should be interpreted together with other structural features, including phosphorylation, fucosylation, sialylation, mannose chain length, and MS/MS-confirmed terminal structures, rather than as an independent predictor of biological efficacy.

To convert complex multi-peak N-glycan profiles into a quantitative metric for quality control, we adopted a strategy analogous to the classic “Z-value” approach used for quality assessment of recombinant human follicle-stimulating hormone (rhFSH). In rhFSH quality control, the Z value represents a weighted sum of glycoforms with distinct charge states (reflecting different numbers of sialic acids) to indicate in vivo half-life. Because the key efficacy-related CQA of rhGBA is the degree of exposed terminal mannose, we proposed a comparable index termed the TME index. The TME index assigns biological weights based on the number of exposed terminal mannose residues in each glycoform and is calculated as follows:

(1) $$TME=\sum_{i=1}^{n}\left(\mathrm{Number}\;\mathrm{of}\;\mathrm{Terminal}\;{\mathrm{Mannose}}_{i}\times \mathrm{Glycan}\;\mathrm{Peak}\;\mathrm{Area}\;{\%}_{i}\right)$$

Based on the above identification and relative quantification results, the calculated TME values for the evaluated lots were 179 for imiglucerase, 266 for velaglucerase alfa, and 272 for velaglucerase beta. The TME index integrates the relative abundance of each glycoform with the number of exposed terminal mannose residues, thereby converting complex multi-peak N-glycan profiles into a single terminal-mannose-related quality descriptor. This metric provides a practical way to compare terminal mannose exposure among rhGBA products manufactured using different glycoengineering strategies and may support batch-to-batch consistency evaluation, process comparability assessment, trend monitoring, and identification of potential glycosylation drift.

The similar TME values of velaglucerase alfa and velaglucerase beta indicate that both products present relatively high levels of terminal mannose exposure, although their glycan distributions are distinct. Velaglucerase beta showed a highly homogeneous Man5-enriched profile, whereas velaglucerase alfa contained more long-chain high-mannose and phosphorylated glycans. These differences suggest that comparable TME values can arise from different glycan architectures and may represent distinct product-specific glycosylation patterns. Therefore, TME should be interpreted as a structural and quality-control descriptor of terminal mannose exposure rather than as an independent predictor of macrophage uptake or clinical efficacy. For comprehensive quality evaluation of rhGBA products, TME should be considered together with phosphorylation-related glycans, mannose-6-phosphate-related structures, mannose chain length, branching features, and MS/MS-confirmed terminal glycan structures.

### 2.3. Elucidation of rhGBA N-Glycan Terminal Structures

After preliminary glycoform assignments, it was observed that molecular-ion information from full-scan MS alone was insufficient to unambiguously determine several glycan structures. Oligosaccharides exhibit pronounced structural isomerism, as glycoforms with identical molecular weights can differ in linkage position, branching architecture, or residue arrangement. Further, some glycoforms generate overlapping fragment-ion clusters upon dissociation, complicating differentiation based solely on accurate mass and retention time. To resolve these ambiguities, detailed CID analysis was performed using a Waters Synapt G2-Si high-resolution mass spectrometer.

In practice, target ions that could not be conclusively assigned by full-scan MS were isolated as precursor ions based on their *m*/*z* values. Controlled fragmentation was induced by applying stepped collision energies, and MS/MS spectra were collected to obtain diagnostic fragment ions, including those indicative of specific modifications. The resulting MS/MS patterns were compared with the literature data and theoretical fragmentation models to confirm structural assignments.

This CID-based fragmentation strategy effectively distinguishes isomeric glycans with identical molecular masses but differing in connectivity, enabling secondary structural verification. The approach enhances the accuracy of N-glycan characterization, supports the construction of a high-confidence rhGBA glycan database, and provides methodological rigor for confirming complex glycan structures.

CID MS/MS was performed on selected precursor ions corresponding to representative chromatographic peaks. By comparing fragmentation patterns and integrating theoretical predictions with the literature evidence [28], structural features, such as core modifications, branching patterns, and terminal groups, were elucidated.

**Figure 2:**
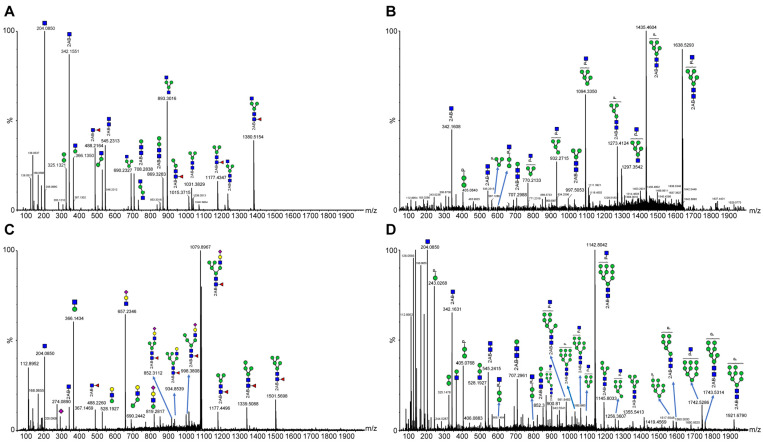
CID MS/MS spectra of four glycoforms requiring structural confirmation. (**A**) MS/MS spectrum of the imiglucerase glycoform at RT 11.48 min (*m*/*z* = 1380.54, z = 1). (**B**) MS/MS spectrum of the imiglucerase glycoform at RT 23.43 min (*m*/*z* = 1638.56, z = 1). (**C**) MS/MS spectrum of the velaglucerase alfa glycoform at RT 34.54 min (*m*/*z* = 1079.40, z = 2). (**D**) MS/MS spectrum of the velaglucerase alfa glycoform at RT 42.94 min (*m*/*z* = 1143.89, z = 2).

For imiglucerase, a precursor ion at *m*/*z* 1380.54 (z = 1) was detected at a retention time of 11.48 min. Its CID spectrum exhibited diagnostic ions consistent with core-fucosylated high-mannose structures, while the absence of the characteristic ion at *m*/*z* 569.21 (HexNAc–Hex–HexNAc + H^+^) indicated that an N-acetylglucosamine (GlcNAc) residue was linked to a terminal mannose, confirming the structure as F(6)Man5 with a terminal GlcNAc residue (Figure 2A). At a retention time of 23.43 min, the precursor ion at *m*/*z* 1638.56 (z = 1) generated phosphorylation-related fragment ions (*m*/*z* 1273.41 and 1435.46), along with ions at *m*/*z* 405.08 and 608.15, confirming the presence of a phosphorylated high-mannose glycan capped with GlcNAc (Man5P–GlcNAc) (Figure 2B). These findings suggest that, in addition to canonical high-mannose glycans, imiglucerase contains a small fraction of phosphorylated glycans derived from residual CHO-cell GNPT/NAGPA pathway activity. Because the ex vivo exoglycosidase process does not cleave phosphodiester linkages, these minor species can persist and be detected by high-resolution MS.

For velaglucerase alfa, a precursor ion at *m*/*z* 1079.40 (z = 2) was detected at 34.54 min. CID analysis produced multiple diagnostic fragments, including galactose-associated ions (*m*/*z* 690.24 and 852.31) and sialic-acid-associated ions (*m*/*z* 292.09, 657.23, and 819.28), corresponding to Hex–HexNAc and Neu5Ac–Gal–GlcNAc fragments. These ions confirmed the presence of a sialylated antenna on the glycan branch. Comprehensive interpretation indicated a core-fucosylated high-mannose glycan F(6)Man5 bearing a terminal Neu5Ac linked via a GlcNAc–Gal motif (Figure 2C). This finding demonstrates that, in addition to high-mannose glycans, velaglucerase alfa contains a minor fraction of terminally sialylated glycans, reflecting expression-system characteristics and glycan-processing depth. Although the abundance (~3%) is low, potential effects of sialylation on pharmacokinetics and immunogenicity warrant consideration. In addition, a precursor ion at *m*/*z* 1143.89 (z = 2) was detected at a retention time of 42.94 min. Its MS/MS spectrum showed a series of characteristic high-mannose ions (*m*/*z* 1355.54, 1580.50, and 1921.68) together with phosphorylation diagnostic ions (*m*/*z* 405.07, Hex–Hex–PO_4_; and *m*/*z* 608.14, Hex–Hex–PO_4_–HexNAc), supporting its assignment as a GlcNAc-capped phosphorylated high-mannose glycan (Man9P–GlcNAc) (Figure 2D). This finding confirms extensive phosphorylation among multi-mannose glycans in velaglucerase alfa, consistent with canonical lysosomal-enzyme mannose-6-phosphate (M6P) biosynthetic pathways involving GlcNAc-1-phosphotransferase (GNPT) capping followed by NAGPA-mediated uncapping, in accordance with the process characteristics of the HT-1080 expression system.

## 3. Instruments and Materials

### 3.1. Instruments and Sources

The following instruments were used: ACQUITY UPLC I-Class system (Waters Corp., Milford, MA, USA); Synapt G2-Si time-of-flight mass spectrometer (Waters Corp.); high-speed refrigerated centrifuge, centrifugal vacuum concentrator, VLP200 vacuum pump, and RVT4104 cold trap (all from Thermo Fisher Scientific, Waltham, MA, USA); pH meter (METTLER TOLEDO, Columbus, OH, USA); temperature-controlled dry block heater (Eppendorf, Hamburg, Germany).

### 3.2. Reagents and Sources

The following reagents were used: guanidine hydrochloride, dithiothreitol (DTT), iodoacetamide (IAM), Tris, hydrochloric acid, dimethyl sulfoxide (DMSO), anhydrous disodium hydrogen phosphate, anhydrous sodium dihydrogen phosphate, sodium cyanoborohydride and 2-aminobenzamide (2-AB) (Sigma-Aldrich, St. Louis, MO, USA); trypsin (Promega, Madison, WI, USA); PNGase F (P0704L and P0709L) and 10% NP-40 (New England Biolabs, Ipswich, MA, USA); formic acid and acetonitrile (Thermo Fisher Scientific, Waltham, MA, USA); leucine enkephalin and sodium iodide (Waters Corp., Milford, MA, USA); glacial acetic acid and ammonia solution (Sinopharm Chemical Reagent Co., Ltd., Shanghai, China); and sodium dodecyl sulfate (SDS), β-mercaptoethanol, absolute ethanol, and methanol (Sinopharm Chemical Reagent Co., Ltd., Shanghai, China).

SPE cartridges and 10 kDa ultrafiltration centrifuge tubes (MilliporeSigma, Burlington, MA, USA); 2-AB Dextran Calibration Ladder, ACQUITY UPLC Peptide BEH C18 (130 Å, 1.7 µm, 2.1 mm × 150 mm), and ACQUITY UPLC Glycan BEH Amide (130 Å, 1.7 µm, 2.1 mm × 150 mm) (all from Waters Corp.).

### 3.3. Mass Calibration and Lock-Mass Correction

Mass calibration and lock-mass correction were performed to ensure mass accuracy during HRMS analysis. Before sample analysis, the Waters Synapt G2-Si Q-TOF mass spectrometer was externally calibrated in positive-ion mode using sodium iodide (NaI) over an *m*/*z* range of 50–2000. The calibration solution was introduced according to the manufacturer’s recommended procedure, and the calibration was accepted only when the residual mass error was less than 1 ppm.

During LC–MS acquisition, leucine enkephalin (LE) was used as the lock-mass compound for real-time mass correction. LE was continuously infused through the LockSpray source, and the protonated ion at *m*/*z* 556.2766 was used as the reference ion in positive-ion mode. Lock-mass scans were acquired every 60 s throughout the analytical run, and the measured *m*/*z* values of sample ions were corrected based on the deviation of the LE reference signal. This combined NaI external calibration and LE lock-mass correction procedure was used to maintain high mass accuracy for glycan assignment.

### 3.4. N-Glycan Profiling

rhGBA samples were processed as follows to release and label N-glycans: In brief, 300 µg of GBA solution was buffer-exchanged three times into 50 mM phosphate buffer (pH 7.5) using a 10 kDa ultrafiltration device (14,000 rpm, 4 °C, 10 min), and approximately 100 µL of retentate was collected for subsequent reactions. An aliquot containing 50 µg of GBA was combined with denaturation buffer, incubated at 80 °C for 20 min, and cooled to room temperature. Subsequently, 8 µL of N-glycosidase reaction buffer, 12 µL of 10% NP-40, and 10 µL of PNGase F were sequentially added. After thorough mixing, the reaction was incubated at 37 °C for 2 h to release N-glycans. The digest was then processed using a 10 kDa ultrafiltration device (14,000 rpm, 15 min) and washed three times to separate the released glycans from protein. The filtrate was vacuum-dried to obtain free N-glycans. Dried glycans were dissolved in 10 µL of ultrapure water, mixed with 6 µL of 2-AB labeling reagent, and incubated at 65 °C for 3 h for derivatization. Samples were washed twice with 95% acetonitrile (14,000 rpm, 5 min), vacuum-dried, and reconstituted in 25 µL of water.

Labeled N-glycans were analyzed by UPLC-FLD-MS. Separation was performed on an ACQUITY UPLC Glycan BEH Amide column with a column temperature of 60 °C, flow rate of 0.4 mL/min, sample temperature of 5 °C, and injection volume of 1 µL. Mobile phase A consisted of 50 mM ammonium formate in water (pH 4.5), and mobile phase B was MS-grade acetonitrile. The gradient program was as follows: 25% A initially; linear increase to 42% A over 60 min; rapid increase to 100% A at 61.5 min and hold until 64.5 min; return to 25% A at 68.1 min and hold until 80 min for column re-equilibration. The FLD was set at 330 nm excitation and 420 nm emission with gain 10. MS detection was conducted on a Waters Synapt G2-Si Q-TOF mass spectrometer in positive-mode ESI. The capillary voltage was 3.5 kV, cone voltage 40 V, and collision energy 4–6 V. Source temperature was 100 °C and desolvation temperature 350 °C. Desolvation gas flow was 600 L/h, cone gas flow 50 L/h, and nebulizer pressure 6.5 bar. The scan range was *m*/*z* 50–2000 with an acquisition rate of 0.5 s/scan.

Data processing (GU matching mode): Retention times of the 2-AB dextran calibration ladder peaks were plotted as the *x*-axis, and corresponding glucose unit (GU) values were used as the *y*-axis to generate a fifth-order calibration equation. Raw data were imported into UNIFI with labeling mode set to 2-AB and the glycan library set to the built-in N-glycan database. The *m*/*z* tolerance was set to 20 ppm, and sample peak assignments were performed based on GU and *m*/*z* values using the fifth-order calibration equation. The full MS screening parameters were: adducts +H and +2H; lock-mass window 0.5 *m*/*z*; reference mass 556.2766; mass error 10 ppm; peak integration region 5–50 min.

### 3.5. CID-Based Elucidation of N-Glycan Terminal Structures

Sample preparation and LC conditions were identical to those used for glycan profiling. UPLC-separated samples were analyzed on a Waters Synapt G2-Si high-resolution Quadrupole-Time-of-Flight (Q-TOF) mass spectrometer in positive-mode ESI. The key parameters were as follows: capillary voltage 1.3 kV; sampling cone 15 V; source temperature 100 °C; desolvation temperature 350 °C; cone gas flow 50 L/h; desolvation gas flow 600 L/h; and nebulizer pressure 6.5 bar. The scan range was *m*/*z* 50–2000 with a scan rate of 0.5 s/scan. MS/MS data were acquired in CID mode with collision energies linearly ramped between 15 and 40 V, depending on precursor charge state and abundance. The instrument was operated in sensitivity mode to enhance detection of low-abundance diagnostic fragments. Based on retention times and *m*/*z* values of four target species, precursor ions at *m*/*z* 1380.54, 1638.56, 1079.40, and 1143.89 were selected for CID. Corresponding collision energy ranges and LC acquisition windows were set to 15–30 V (10–15 min), 20–40 V (22–28 min), 20–40 V (33–37 min), and 25–45 V (40–45 min), respectively, to obtain characteristic fragment-ion information.

### 3.6. Method Validation

The developed N-glycan profiling method was validated in terms of specificity, linearity, repeatability, intermediate precision, accuracy, limit of detection (LOD), and limit of quantification (LOQ). Validation was performed using representative rhGBA samples, reference/sample preparations, and formulation buffer according to a predefined experimental design.

Specificity was evaluated by analyzing the formulation buffer and sample preparations. The chromatograms were examined to determine whether the formulation buffer produced any interfering chromatographic peaks within the retention-time window of 5–55 min.

Linearity was assessed by preparing samples at 50%, 80%, 100%, 150%, and 180% of the nominal reference standard amount. Each concentration level was prepared in triplicate. The peak area of the main glycan component, Man5, was used as the representative response, and linear regression analysis was performed to evaluate the relationship between sample amount and detector response.

Repeatability was evaluated by preparing six samples in parallel on the same day. The relative abundances of Man5 and total phosphate-related oligosaccharides were calculated for each preparation. In addition, the relative retention times of the three major peaks were assessed to evaluate chromatographic consistency.

Intermediate precision was evaluated using 12 samples analyzed on different days. The relative abundances of Man5 and total phosphate-related oligosaccharides were calculated. The relative retention times of Man4 and F(6)Man5 versus Man5 were further assessed to evaluate retention-time reproducibility under intermediate precision conditions. In this study, relative retention time (RRT) was used for peak tracking and chromatographic reproducibility assessment during method validation, whereas glucose unit (GU) calibration was used as a retention-time standardization approach to support N-glycan assignment.

The LOD and LOQ were evaluated using low-level sample preparations. The selected low-abundance glycan peak, represented by M3, was used as the representative analyte. The LOD and LOQ were estimated based on signal-to-noise ratios of approximately 3 and 10, respectively. For LOQ assessment, two low-level sample preparations were analyzed, and the signal-to-noise ratio and peak-area reproducibility of the selected peak were evaluated.

Accuracy was evaluated by preparing samples at 50%, 100%, and 150% of the nominal reference standard amount. Each concentration level was prepared in triplicate. The measured values of Man5% and total mannose-related oligosaccharide% were compared with the corresponding theoretical values, and mean percent recoveries were calculated.

## 4. Conclusions

This study provides a systematic and comprehensive characterization of N-glycan structures on rhGBA. By integrating method development and validation for glycan profiling with CID-based terminal-structure elucidation, we delineated the multidimensional landscape of GBA glycosylation. A sensitive, high-resolution UPLC-FLD-MS method was successfully established and validated, demonstrating strong reliability and complementarity for both qualitative and relative quantitative analyses. The method enables detailed comparison of glycoform composition and abundance across rhGBA products and, through CID MS/MS, allows confident structural confirmation of complex glycans. Collectively, these findings establish a robust analytical framework for GBA quality control and structural characterization.

The results reveal pronounced differences in N-glycosylation patterns among rhGBA products derived from distinct manufacturing processes. As a CHO-derived enzyme subjected to ex vivo glycosidase trimming, imiglucerase exhibits a highly processed glycan profile dominated by F(6)Man3. This simplified terminal architecture, combined with elevated core fucosylation, maximizes exposure of core mannose residues and may enhance mannose receptor affinity [29]. Nevertheless, due to the complexity of the trimming process, a degree of microheterogeneity persists, likely attributable to incomplete glycan cleavage.

In contrast, velaglucerase alfa, expressed in a human fibroblast-derived cell line, employs the α-mannosidase I inhibitor kifunensine to prevent glycan maturation, preserving long-chain high-mannose structures, with Man9 constituting approximately 40%. Although Feinberg et al. observed that long-chain glycans may experience steric constraints when binding a single receptor site [29], Van Patten et al. [12] emphasized the advantage of multivalency: extended high-mannose structures can engage multiple carbohydrate-recognition domains on MR simultaneously. This mechanism explains the approximately 2–2.5-fold higher macrophage uptake rate of velaglucerase alfa compared with imiglucerase [13]. Further, phosphorylated glycoforms (e.g., Man9P–GlcNAc) are consistent with lysosomal enzyme characteristics and may contribute synergistically to lysosomal targeting. The detection of minor sialylated F(6)Man5 species underscores the method’s sensitivity and indicates that potential effects of sialylation on pharmacokinetics and immunogenicity in VPRIV^®^ should be considered.

Velaglucerase beta represents a next-generation glycoengineering approach. By employing CRISPR–Cas9-mediated knockout of the GnT1 gene in CHO cells, glycan processing is halted at the high-mannose stage. This genome-level modification yields a high degree of structural uniformity, with Man5 representing over 70% of total glycans, producing a predominantly single-glycoform profile. Such reduced heterogeneity may correlate with a lower risk of in vivo immunogenicity.

Together, these observations highlight the profound influence of expression systems and process conditions on glycan modifications, providing plausible mechanistic explanations for differences in delivery efficiency and clinical outcomes among products. Terminal-structure elucidation further clarifies linkage arrangements and exposed residues, showing that mannose distribution and phosphorylation are key determinants of lysosomal targeting and cellular uptake. Additionally, fucosylation and sialylation in certain glycoforms may affect immunogenicity and molecular stability. These findings emphasize the importance of precise control over glycosylation and reinforce that glycan features should be stringently monitored as core CQAs during product development and comparability studies.

To enhance QC efficiency for oligosaccharide profiling of recombinant enzymes, we recommend standardized reporting of glycan profiles using the TME index. This metric reduces interpretational variability caused by fluctuations in individual glycoforms, promotes consistency and objectivity in data interpretation, and improves sensitivity for detecting process drift and quality trends, thereby strengthening ongoing process verification and performance qualification.

This study provides a systematic analytical solution for N-glycan structural characterization of recombinant GBA and supports the future establishment of product specifications and potential clinical optimization. However, applicability of the proposed method to other ERT enzymes (e.g., agalsidase, alglucosidase, and laronidase) remains to be assessed. Further, because phosphorylated glycans exhibit lower ionization efficiency in positive-ion mode, their detection limits may be relatively higher. With continued advancements in glycomics, MS platforms, and data-processing algorithms, N-glycan structural elucidation is expected to play an increasingly critical role in biologics development, quality control, and clinical translation, supporting more-reliable and precise therapies for rare diseases such as Gaucher disease.

## Figures and Tables

**Table 1:** Method validation results for the rhGBA N-glycan profiling assay.

Validation Item	Validation Result
Specificity	No obvious chromatographic peaks were observed for the formulation buffer between 5 and 55 min.
Linearity	For the peak area of the main component Man5, the linear correlation coefficient was R^2^ = 0.9994.
Repeatability	Across six samples processed in parallel, the RSDs of Man5% and total phosphate-related oligosaccharide% were 0.01% and 2.34%, respectively. The relative retention times of the three major peaks were within 0.75–0.95 and 0.95–1.15.
Intermediate precision	Across 12 samples analyzed on different days, the RSDs of Man5% and total phosphate-related oligosaccharide% were 0.33% and 3.49%, respectively. The relative retention times of Man4 and F(6)Man5 versus Man5 were within 0.75–0.95 and 0.95–1.15.
LOD/LOQ	Man3 was selected as the representative low-abundance glycan. The Man3 peak showed S/N = 3.7 at a protein loading amount of 5 μg and S/N = 11.8 at a protein loading amount of 15 μg.
Accuracy	For Man5%, the mean percent recoveries (measured/theoretical) were 101%, 100%, and 96%.

**Table 2:** Mass increments associated with addition of structural units to an oligosaccharide.

Name	Chemical Formula	Monoisotopic Mass (Da)	ΔM Upon Attachment to an Oligosaccharide (Da)
Mannose (Hex)	C_6_H_12_O_6_	180.0634	+162.0528
Fucose (dHex)	C_6_H_12_O_5_	164.0685	+146.0579
N-acetylglucosamine (GlcNAc)	C_8_H_15_NO_6_	221.0899	+203.0794
Sialic acid (Neu5Ac)	C_11_H_19_NO_9_	309.1060	+291.0954
Phosphate group (HPO_3_)	HPO_3_	79.9663	+79.9663

**Table 3:** Glycoform distribution and terminal mannose counts of three rhGBA products.

Sample	Glycan		RT (min)	Peak Area%	Observed *m*/*z*	Expected *m*/*z*	ppm	Terminal Mannose
	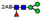	F(6)Man3	9.07	50.20	1177.4566	1177.4617	4.33	2
	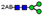	Man3	7.18	16.47	1031.4090	1031.4038	5.04	2
	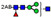	F(6)Man3 NAc a	11.48	9.57	1380.5373	1380.5411	2.75	1
	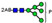	Man6P a	29.46	5.15	1597.5315	1597.5286	1.82	2
	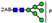	Man6P b	28.61	3.73	1597.5315	1597.5286	1.82	2
	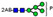	Man5 P	24.27	3.44	1435.4721	1435.4758	2.58	2
	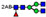	F(6)Man3 NAc2	14.51	2.00	1583.6174	1583.6205	1.96	0
**Imiglucerase**	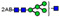	Man5 NAc	19.28	1.55	1558.5816	1558.5888	4.62	2
	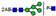	Man5 P NAc	23.43	1.47	1638.5482	1638.5552	4.27	2
	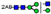	Man4 NAc	13.94	1.08	1396.5315	1396.5360	3.22	1
	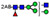	F(6)Man4 NAc	16.66	0.96	1542.5896	1542.5939	2.79	1
	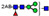	F(6)Man3 NAc b	11.92	0.81	1380.5373	1380.5411	2.75	1
	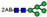	Man5	16.31	0.77	1355.5047	1355.5095	3.54	3
	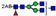	F(6)Man3 NAc3	17.12	0.70	1786.7166	1786.6998	9.40	0
	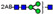	Man3 NAc	9.76	0.46	1234.4827	1234.4800	2.19	1
	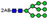	Man9 a	37.15	39.65	1002.3639 *	1002.3640 *	0.10	3
	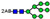	Man8 a	32.31	8.03	1841.6773	1841.6679	5.10	3
	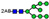	Man8 b	32.94	7.94	1841.6773	1841.6679	5.10	3
	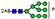	Man9 P	43.85	7.25	1042.3480 *	1042.3472 *	0.77	2
	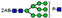	Man9 P NAc	42.94	6.69	1143.8822 *	1143.8869 *	4.11	2
	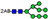	Man8 c	31.90	5.67	1841.6773	1841.6679	5.10	3
	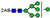	Man7 a	27.62	3.53	1679.6089	1679.6151	3.69	3
**Velaglucerase α**	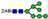	Man7 b	26.74	3.31	1679.6089	1679.6151	3.69	3
	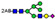	F(6)Man5 NAc Gal SA	34.54	2.87	1079.4095 *	1079.4011 *	7.78	2
	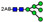	Man6	21.83	2.04	1517.5612	1517.5623	0.72	3
	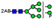	Man8 P a	38.74	1.89	1921.6418	1921.6343	3.90	2
	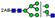	Man8 P NAc a	39.66	1.71	1062.8609	1062.8605	0.38	2
	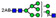	Man8 P NAc b	40.22	1.42	1062.8609	1062.8605	0.38	2
	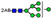	Man7 c	27.45	1.22	1679.6089	1679.6151	3.69	3
	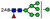	F(6)Man7	29.27	1.15	1825.6906	1825.6730	9.64	2
	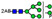	Man8 P b	39.22	1.10	1921.6418	1921.6343	3.90	2
	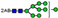	Man9 b	37.79	1.06	1002.3639 *	1002.3640 *	0.10	3
	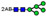	Man5 a	16.39	72.65	1355.5047	1355.5095	3.54	3
	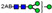	Man4	10.94	9.51	1193.4527	1193.4566	3.27	2
	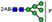	Man6 P b	29.51	4.85	1597.5315	1597.5286	1.82	2
	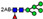	F(6)Man5	18.99	3.95	1501.5713	1501.5674	2.60	3
	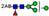	F(6)Man4	13.31	1.61	1339.5081	1339.5145	4.78	2
**Velaglucerase β**	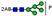	Man5 P a	24.32	1.53	1435.4721	1435.4758	2.58	2
	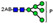	Man6 P c	30.53	1.12	1597.5315	1597.5286	1.82	2
	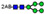	Man5 b	17.00	0.74	1355.5047	1355.5095	3.54	2
	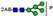	Man5 P b	27.21	0.77	1435.4721	1435.4758	2.58	2
	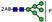	Man6 P a	28.68	0.48	1597.5315	1597.5286	1.82	2
	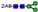	Man3	6.69	0.36	1031.4093	1031.4038	5.33	2

Note: Glycan structures are presented using abbreviated notation in this table. For example, Man5 denotes Man5GlcNAc2. The letters a, b, and c following glycan names indicate different isomeric forms. The *m*/*z* values marked with an asterisk (*) indicate doubly charged ions.

## Data Availability

The original contributions presented in this study are included in the article. Further inquiries can be directed to the corresponding authors.

## Abbreviations

The following abbreviations are used in this manuscript:

2-AB	2-Aminobenzamide
CHO	Chinese hamster ovary
CID	Collision-induced dissociation
CQA	Critical quality attribute
CID-MS/MS	Collision-induced dissociation tandem mass spectrometry
ERT	Enzyme replacement therapy
ESI	Electrospray ionization
GBA	Glucocerebrosidase
rhGBA	Recombinant human glucocerebrosidase
SDS	Sodium dodecyl sulfate
FLD	Fluorescence detection
GU	Glucose unit
GD	Gaucher disease
GlcCer	Glucosylceramide
GlcNAc	N-acetylglucosamine
Hex	Hexose
GnT1	N-acetylglucosaminyltransferase I
GNPT	GlcNAc-1-phosphotransferase
HILIC	Hydrophilic interaction liquid chromatography
HT-1080	Human fibrosarcoma cell line HT-1080
HRMS	High-resolution mass spectrometry
LC	Liquid chromatography
LE	Leucine enkephalin
LOD	Limit of detection
LOQ	Limit of quantification
M6P	Mannose-6-phosphate
TME	Terminal mannose exposure
UPLC	Ultra-performance liquid chromatography
